# A Framework for Malicious Traffic Detection in IoT Healthcare Environment

**DOI:** 10.3390/s21093025

**Published:** 2021-04-26

**Authors:** Faisal Hussain, Syed Ghazanfar Abbas, Ghalib A. Shah, Ivan Miguel Pires, Ubaid U. Fayyaz, Farrukh Shahzad, Nuno M. Garcia, Eftim Zdravevski

**Affiliations:** 1 Al-Khwarizmi Institute of Computer Science (KICS), University of Engineering & Technology (UET), Lahore 54890, Pakistan; ghazanfar.abbas@kics.edu.pk (S.G.A.); ghalib@kics.edu.pk (G.A.S.); ubaid.fayyaz@kics.edu.pk (U.U.F.); farrukh.shahzad@kics.edu.pk (F.S.); 2 Instituto de Telecomunicações, Universidade da Beira Interior, 6200-001 Covilhã, Portugal; ngarcia@di.ubi.pt; 3 Computer Science Department, Polytechnic Institute of Viseu, 3504-510 Viseu, Portugal; 4 UICISA: E Research Centre, School of Health, Polytechnic Institute of Viseu, 3504-510 Viseu, Portugal; 5 Faculty of Computer Science and Engineering, University Ss Cyril and Methodius, 1000 Skopje, North Macedonia; eftim.zdravevski@finki.ukim.mk

**Keywords:** Internet of Things (IoT), IoT healthcare systems, healthcare monitoring, machine learning, securing healthcare systems, IoT healthcare dataset, IoT traffic generator, IoT flock, healthcare security, intrusion detection

## Abstract

The Internet of things (IoT) has emerged as a topic of intense interest among the research and industrial community as it has had a revolutionary impact on human life. The rapid growth of IoT technology has revolutionized human life by inaugurating the concept of smart devices, smart healthcare, smart industry, smart city, smart grid, among others. IoT devices’ security has become a serious concern nowadays, especially for the healthcare domain, where recent attacks exposed damaging IoT security vulnerabilities. Traditional network security solutions are well established. However, due to the resource constraint property of IoT devices and the distinct behavior of IoT protocols, the existing security mechanisms cannot be deployed directly for securing the IoT devices and network from the cyber-attacks. To enhance the level of security for IoT, researchers need IoT-specific tools, methods, and datasets. To address the mentioned problem, we provide a framework for developing IoT context-aware security solutions to detect malicious traffic in IoT use cases. The proposed framework consists of a newly created, open-source IoT data generator tool named IoT-Flock. The IoT-Flock tool allows researchers to develop an IoT use-case comprised of both normal and malicious IoT devices and generate traffic. Additionally, the proposed framework provides an open-source utility for converting the captured traffic generated by IoT-Flock into an IoT dataset. Using the proposed framework in this research, we first generated an IoT healthcare dataset which comprises both normal and IoT attack traffic. Afterwards, we applied different machine learning techniques to the generated dataset to detect the cyber-attacks and protect the healthcare system from cyber-attacks. The proposed framework will help in developing the context-aware IoT security solutions, especially for a sensitive use case like IoT healthcare environment.

## 1. Introduction

The Internet of things (IoT) refers to real-world objects having communicative and cognitive capability using smart devices. IoT is a tremendous communication paradigm where the plethora of heterogeneous devices will connect and talk to each other. These communication devices will play an essential role in the life of human beings. IoT is creating a revolutionary impact in the world of technology and the social life of people. Over time, IoT devices are overgrowing. According to recent statistics, the number of IoT devices connected worldwide was 23.14 billion in 2018 [1]. Furthermore, it is estimated that by 2025 there will be 75.44 billion IoT connected worldwide [1]. IoT footprints have been identified in various domains such as manufacturing, agriculture, transportation, electric grid, healthcare, among others [2,3,4]. In an IoT-based healthcare system, security is the primary concern as the data is directly related to human beings. An intensive care unit (ICU) is a special and critically operational department of a hospital where specialized treatment is given to the patients who require critical medical care. Usually, the patients who are acutely unwell or injured severely and require continuous medical care are admitted to the ICU. The equipment and devices concerned in the ICU play a vital role in keeping the patient alive and healthy. In such a scenario, any communication breakdown due to a cybersecurity breach may cause severe effects on a patient’s life and even death in some instances. Therefore, an attack-proof IoT-based healthcare system is a primary need of the hour. Thus, providing an IoT-based healthcare system with secure and robust data communication among medical sensors and actuators is obligatory.

So far, a lot of work has been done to automate the patients’ monitoring and assist the medical professional in remotely assessing the patient’s health status [5,6,7,8,9]. To preserver patients’ privacy, some approaches rely on fog computing to perform some of the computation on the edge, so that identifying information is not sent to the cloud [10]. However, the cyber-attacks on IoT healthcare systems have become the primary focus of the intruders [11]. It is reported that more than 90% of the healthcare systems have been the victim of the cyber-attacks. In 2017, White House intelligence reported that the healthcare sector has become attractive for cyber-criminals [12]. Moreover, during the COVID-19 era, the cyber-attacks exponentially surged across the spread of COVID-19 [13]. The cyber-criminals have now started targeting the IoT healthcare services and systems actively [13,14].

The confidentiality and integrity of the data have utmost importance in IoT healthcare environments [15]. Any data breach or data tampering in healthcare systems can revoke serious health hazards to patients. Likewise, any change to the patient’s medical record may lead to accidental death if unnoticed by the professional medical [11]. Furthermore, the cyber-attacks on IoT healthcare systems can cause critical disruption in the medical treatments [11,14]. For example, if a hacker gains access to an infusion pump and then changes the infusion pump’s configuration in such a way that it starts releasing a large amount of insulin to a patient, which can cause severe hypoglycemia. Similarly, suppose a cybercriminal gains access to a pacemaker and either slows down the heart rate or speeds up the heart rate. In that case, severe bradycardia or tachycardia may instantly happen to the patient, ultimately leading to death. Henceforth, the security of healthcare systems has become the most significant concern of this age.

The IoT devices are resource constraint because of the limited amount of memory, computational capacity, and power [16,17]. The traditional security solutions are not adequate for efficiently detecting the malicious attacks in IoT environment due to the distinct features of IoT devices [17]. Therefore, it is very challenging to design a security mechanism for IoT devices. The manufacturing industries are producing a massive amount of IoT products to create low-cost IoT devices in a short period [3,18]. Due to the race to the market for capturing the market as earlier as possible, the manufacturers are giving less importance to the device security [3]. Moreover, the IoT device manufacturers also create back doors to access the machine remotely or use that device for their malicious intent. Most of the consumer-deployed IoT devices are connected to the network without any security defense line [19]. Therefore, IoT devices can be compromised easily. Therefore, the security of IoT devices has become a significant concern nowadays.

The traditional security mechanisms are deployed at two levels, i.e., network-level or host-level [20]. In the current Internet era, the host-level security approach is considered more secure than the network level security approach [16]. As IoT devices are low-powered and resource-constrained, host-level security mechanisms cannot be deployed. Therefore, the network-based security mechanism is preferable for IoT devices compared to the host-based security system [16,19].

An intrusion detection system (IDS) is a compulsory defense line among the existing security mechanisms responsible for identifying malicious activities in the network. It is also classified into network-based and host-based IDS. The network-based IDS scrutinizes network events, while the host-based IDS scrutinizes host events. The network-based IDS needs to be trained in IoT to protect the IoT networks and network from cyber-attacks. The IDS are trained either on live traffic or on a recorded network traffic dataset. The IDS training on live traffic is an expensive and time-consuming procedure. However, the training of IDS on an appropriate network traffic dataset is a fast and low-cost procedure. Thus, having an appropriate IoT dataset for the IDS training and testing is a crucial need. Some approaches, such as in [21], rely on deep learning models for feature extraction, aiming to perform intrusion detection in network traffic. Although the existing IDS technology is well entrenched however, it is inadequate for the IoT devices and network [22,23] due to the limited processing and storage capacity. Moreover, the commonly used IoT protocols like MQTT, and Constrained Application Protocol (COAP), are not supported by the traditional IDS solutions. Therefore, it the need of the hour to develop IoT-supported IDS.

Recently, the authors of [4] surveyed the security requirements and various cyber-attacks of the wireless sensor network (WSN) and IoT environment. The authors conducted a detailed review of various security solutions proposed for WSN and IoT. Their study revealed that data generated by IoT devices in an IoT environment varies from use case to use case, e.g., smart healthcare, smart transportation, etc. Thus, the traditional security solutions, mechanisms, need to be tailored for IoT use case requirements. Thereby, the researchers need IoT-specific tools, methods, and datasets to enhance security for IoT devices and networks. The training and testing of IDS require an efficient IoT traffic dataset that contains both standard and malicious IoT traffic. Only a few researchers are working to produce a suitable IoT dataset to test and evaluate the IoT-supported IDS. In this work, we propose the development of IoT context-aware security solutions to detect malicious traffic in IoT healthcare environments. To efficiently develop the IoT context-aware security solutions, the proposed framework consists of a newly created, open-source IoT data generator tool, named IoT-Flock [16,24], for generating IoT use case-specific traffic.

Although the existing IDS technology is well entrenched, it is inadequate for the IoT devices and network [22] due to the limited processing and storage capacity. Thus, the traditional security solutions are not adequate for efficiently detecting the malicious attacks in IoT environment due to the distinct features of IoT devices such as scalability, processing, memory, and power resource limitations, etc. [17]. Therefore, it is vital to develop IoT-supported IDS. The training and testing of IDS require an efficient IoT traffic dataset that contains both normal and malicious IoT traffic. Only a few researchers are working to produce a suitable IoT dataset to test and evaluate the IoT-supported IDS. To this aim, we generated a real-time IoT traffic dataset for the development of IoT-based IDS specifically in ICU-context. Additionally, the proposed framework provides an open-source utility for converting the captured traffic generated by IoT-Flock [16,24] into an IoT dataset. Finally, by applying different machine learning techniques over the generated IoT dataset, we demonstrate how the proposed framework can be used to develop an AI-based cyber-security solution to secure and protect the IoT healthcare systems from cyber-attacks. The proposed framework will help in developing context-aware IoT security solutions, especially for the sensitive healthcare environment.

This paper presents a framework for developing IoT context-aware security solutions to detect malicious traffic in IoT use cases, especially for the IoT healthcare environment. The proposed framework consists of an open-source IoT traffic generator tool and an IoT use case dataset to ease the research community. To the best of our knowledge, this is the first time that an open-source IoT traffic generator tool is introduced. It can also generate both the normal and malicious IoT traffic based on two IoT application layer protocols: Message Queuing Telemetry Transport (MQTT) and COAP. So far, no IoT traffic generator tool is capable of generating malicious IoT traffic. We used the IoT traffic generator tool to create an attacking network and a typical network of IoT devices for generating the network traffic to capture the packets and a complete payload for the dataset. We designed a real-life ICU use case in the IoT traffic generator tool. We created the data profiles and time profiles of the ICU devices by analyzing real-time value ranges for generating the real-time IoT traffic. We extracted the network layer features and introduced such an extensive set of application-layer features and payload features. They have not been done before from an IoT perspective to generate a widespread and reliable IoT traffic dataset for IoT-supported IDS training and testing. Finally, we demonstrated the proposed framework’s applicability and the generated dataset for developing the IoT context-aware security solutions using six commonly used machine learning algorithms.

The rest of the paper is structured as follows. Section 2 presents the existing IDS datasets’ literature review. Section 3 describes the framework proposed for developing the IoT context-aware security solutions and a detailed overview of our IoT traffic generator tool, IoT healthcare use case conceived for generating the dataset, traffic generation scenario, data capturing procedure used for recording the generated traffic, and the features of the dataset. We discussed the results of the proposed framework in Section 4; it ends with the analysis of generated dataset using machine learning techniques. Last, Section 5 concludes the paper.

## 2. Literature Review

The rapid growth of IoT technology has revolutionized many areas of life by familiarizing the concept of smart cities, smart health, smart wearables, smart industries, smart agriculture, among others. IoT-based health monitoring systems are becoming smarter day by day. In recent years, various IoT-based smart health monitoring systems have been proposed. However, IoT is still in its infancy in the security of healthcare systems. The conventional network security solutions cannot be used for IoT-based healthcare systems due to the resource constraint property of IoT devices and different security level requirements [4,17]. Furthermore, the data generated by IoT devices in an IoT environment varies from use case to use case, e.g., smart healthcare, smart home, among others. Thus, the traditional security solutions, mechanisms, needs to be tailored with respect to IoT use case requirements [4]. Finally, there is a strong need for obstructing malicious activity from accessing the network of resource-constrained IoT-based medical devices.

So far, many researchers have worked and proposed different solutions to secure the IoT healthcare systems from the cyber-attacks. Rughoobur et al. [15] proposed a framework to detect the replay attacks on battery-dependent IoT healthcare devices. Their proposed solution analyzes the unique device id, timestamps, and battery depletion behavior to detect and mitigate the replay attacks on battery-dependent IoT healthcare devices. Rathore et al. [17] introduced an extreme learning machine (ELM) based semi-supervised Fuzzy C-Means method to detect the cyber-attacks in fog-based IoT environments. The authors used ELM to timely and efficiently detect the cyber-attacks and used Fuzzy C-Means to mitigate the dataset label issues. Likewise, Carta et al. [20] introduced a feature engineering technique to efficiently detect the anomalies in order to improve the performance of traditional IDSs. Similarly, Alrashdi et al. [25] proposed a framework for detecting the malicious in fog-based IoT healthcare system. The authors used an ensemble of online sequential ELM to detect malicious attacks like man-in-the-middle, DDoS, etc. attacks in a fog-based smart home equipped with a remote patient monitoring system.

As discussed earlier that in the current era, security is the primary concern of IoT. Firewalls, IDS, and intrusion prevention systems (IPS) are the primary security shields to protect the devices and network from cyber-attacks. Most of the firewalls filter the normal and suspicious traffic based upon the static defined rules. However, IDS and IPS filter the intrusive attempts using artificial intelligence (AI) techniques and are thus more reliable and effective than the predefined static rules. The IDS and IPS are trained and tested using malicious and benign network traffic datasets. Two approaches collect these datasets: either by using real systems to generate suspicious and normal network traffic or using the traffic generators. The traffic generators are the tools that mimic the real-time network traffic.

The training and testing of the IDS and IPS using the network traffic dataset generated by real-time systems represent a costly and difficult job. However, this dilemma can be untangled by generating the dataset through a traffic generator tool. No matter, the present IDS technology is relatively mature, but these are inadequate for IoT Systems [22,23]. As previously discussed, the main reason behind this is the limited processing and storage capacity of the IoT nodes, which is the main challenge for host IDS [16,17]. Another crucial factor is that the communication protocols (like CoAP, MQTT, etc.) IoT devices use are not employed in the traditional network as different protocols carry different vulnerabilities and demands for IDS [23]. This work’s primary focus is to generate a vast and reliable IoT network traffic dataset to develop IDS specifically for the IoT-based ICU environment. Any security breach in such a scenario may either cause a severe effect on the patient or even death in some instances [11].

There exist some state-of-the-art datasets that are used for the training and testing the IDS and IPS for the conventional network and IoT network. The network traffic datasets proposed during the past few years are being used widely until this age. Some of them are realistic datasets, i.e., generated using real-time systems, while some are simulated datasets, i.e., generated through simulation tools. Some of the state-of-the-art datasets are DARPA [26], KDD-99 [27], NSL-KDD [28], DEFCON [29], LBNL [30], CAIDA [31], UNIBS [32], ISCX [33], and UNSW-NB15 [34].

DARPA [26] is the pioneer dataset proposed in 1998 at MIT Lincoln Laboratory. This dataset was developed for the assessment of IDS, and it contains recorded data of seven weeks. It includes the data of emails, FTP, Telnet, IRC, and SNMP-related events. Different types of attacks like denial of service (DoS) attacks, remote to local (R2L) attacks and user to remote (U2R) attacks simulated using Windows, UNIX platforms. However, this dataset does not contain real-time traffic and has some irregularities like the absence of false positives [35].

The KDD-99 [27] dataset was produced using DARPA [26] to inspect the IDS explicitly designed for detecting the inbound attacks. It contains DoS attacks like pod-DoS, smurf-DoS, Neptune-DoS, and buffer overflow attacks. This dataset had a significant problem of imbalanced learning as it consists of 80% attack traffic. Later on, NSL-KDD [28] was generated to fix the issues in KDD-99 [27]. However, it is also outdated as it does not the current standard and attack events.

The DEFCON [29] dataset has two versions—DEFCON-8 and DEFCON-10, recorded in 2000 and 2002, respectively, during a capture flag (hacking and anti-hacking) competition. DEFCON-8 includes the port scanning attacks and buffer overflow attacks, while DEFCON-10 includes attacks related to port scan, malicious packets, and FTP attacks.

LBNL [30] dataset was produced by gathering the inbound, outbound, and routing traffic of two edge routers in 2004. It was collected at a medium-sized site, contains complete header files only. It suffers from heavy anonymization.

CAIDA [31] dataset contains only traffic header without the payload and collected from the Internet backbone of a large-scale enterprise. It includes specific attacks such as DDoS attacks. The recorded dataset was not further processed to generate new features that could improve the distinction between normal and malicious traffic. Moreover, it is unlabeled, so not beneficial for the direct performance assessment of IDS as it needs the preprocessing for labeling.

The UNIBS [32] dataset was captured through tcpdump of the router with the traffic flow of twenty workstations. Only the DoS attack was focused. ISCX [33] was produced using real network configurations in 2012. A group of people was involved in simulating real network traffic. It is a labeled dataset with different attack scenarios. It contains two profiles: the Alpha-profile carried out at multi-stage attack scenarios and the Beta-profile carried out regular traffic. It includes full packet payloads of different protocols like HTTP, FTP, SMTP, and others. However, it does not contain HTTPS, which is 70% of current age traffic. The UNSWNB-15 [34] dataset was collected by generating the IXIA storm to generate malicious and regular traffic in a commercial penetration testing environment. Bot-IoT [36] is a dataset that contains the simulated IoT network traffic and along with different types of attacks.

In the existing literature, most of the studies use some of the above-mentioned datasets for developing security solutions for the IoT environment. However, as we discussed earlier, the traffic generated by IoT devices in an IoT environment varies from use case to use case, e.g., smart healthcare, smart home, etc. Thus, the traditional security solutions (mechanisms) need to be tailored with respect to IoT use case requirements [4]. Moreover, due to the limited processing and storage capacity, application-layer protocols, among others, the traditional security solutions are not adequate for efficiently detecting the malicious attacks in IoT environment [17]. Therefore, it the need of the hour to develop IoT-supported IDS by using the IoT use case related traffic.

To our best knowledge, among the above-mentioned datasets, Bot-IoT [36] is the only publicly available IoT dataset that contains both normal and malicious traffic. The Bot-IoT [36] dataset includes the IoT traffic generated by the normal and attacking virtual machines for the five IoT scenarios provoked with three types of probing attacks, DoS attacks, and information theft attacks. Although the Bot-IoT [36] dataset contains approximately seventy-two-billion instances, only one IoT-specific protocol is considered, i.e., MQTT. While using our developed traffic generator tool, i.e., IoT-Flock [16,24], we can create almost any IoT use case, add normal and malicious devices into it with respect to the use case, and generate the traffic of two major IoT application-layer protocols, i.e., MQTT and CoAP.

## 3. Proposed Framework

**Figure 1:**
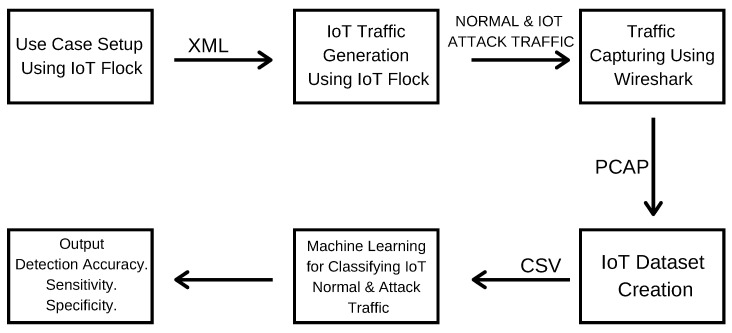
Framework for research in IoT health care security.

From the IoT use case traffic generation to the development of context-aware IoT security solution for malicious traffic detection in IoT healthcare use case, the proposed framework consists of five major stages as illustrated in Figure 1. These modules include use case setup, IoT normal and attack traffic generation, IoT traffic capturing, IoT dataset creation, and machine learning (ML) model development.

The proposed framework starts from designing an IoT use case and then executing that use case to generate real-time IoT traffic. The generated traffic is then captured to create an IoT dataset for training the ML models. Finally, ML models are trained and tested by applying different ML techniques to efficiently detect the malicious network traffic in the underlying IoT use case.

### 3.1. IoT Use Case Generator

**Figure 2:**
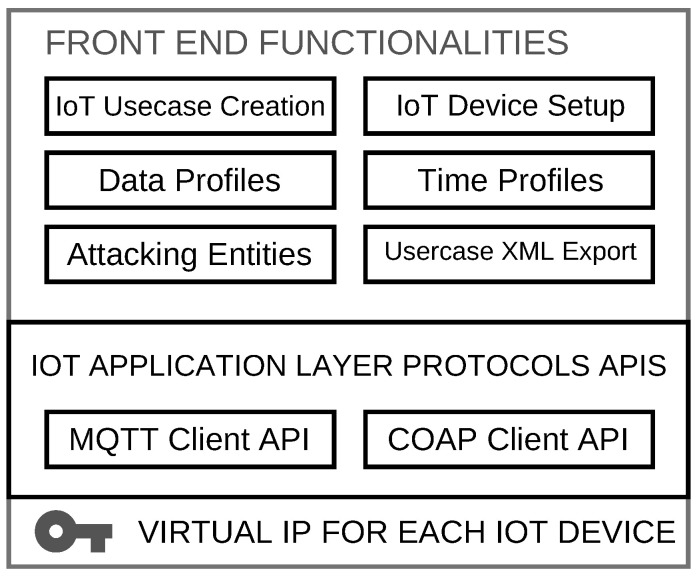
Basic architecture of the IoT-Flock [16,24] tool developed for IoT traffic generation.

IoT use case generator is the first module of the proposed framework, consisting of our recently developed, open-source IoT traffic generator tool, i.e., IoT-Flock [16,24]. The IoT-Flock [16,24] tool can generate both the normal and attack traffic of IoT-based devices in any use case. With the IoT-Flock [16,24], one can easily create IoT use cases according to his/her needs and generate IoT traffic for different use cases to create the traffic for hundreds of IoT devices in a real-time live network by using a single physical machine. The basic architecture of the IoT-Flock [16,24] is shown in Figure 2.

The IoT-Flock [16,24] tool has the following distinct features as compared to the other commercially or publicly available traffic generator tools:

1. IoT-Flock [16,24] is an open-source tool with easily understandable and extendable code.
2. IoT-Flock [16,24] can create real-time IoT use cases with the support of many devices that can be added.
3. Most of the open-source and commercially available traffic generator tools do not support creating the attacking entities. Simultaneously, IoT-Flock [16,24] allows the researchers to develop both standard and attacking devices in the same use case and generate its traffic. Thus, the generated traffic contains both standard and attach patterns, which can help design IDS and IPS better.
4. IoT-Flock [16,24] provides the support to export the designed use case into XML and provides the support to import the XML generated whether by using IoT-Flock [16,24] or some other third-party tool. It can motivate the researchers to create more user-friendly use cases, export them to XML, and run it using the IoT-Flock [16,24] console application for IoT traffic generation.
5. IoT-Flock [16,24] is capable of generating the latest MQTT, COAP specific attacks. That is not supported yet by any other open-source IoT traffic generator tools. Users can select an attack type and create a malicious device, thus can quickly generate malicious traffic.

IoT-Flock [16,24] has two working modes, i.e., GUI mode and Console mode. The GUI mode is designed to provide a user-friendly environment for creating IoT use cases. One can make any IoT use case and add any number of devices into it. The devices added to the use case can mimic the real-time devices. A user can create a single IoT device or multiple IoT devices at a time. For creating a single IoT device or multiple IoT devices, a user will have to provide the relevant functional and non-functional information about the device. The functional information defines the working behavior of an IoT device. It includes the information about the device type (i.e., normal or malicious), protocol, data profile, time profile, commands, and controls (i.e., subscribe, publish topic in MQTT case during getting, post control in case of COAP). On the other hand, the user’s non-functional information is provided to distinguish an IoT device from the other IoT devices. The functional and non-functional information fields are briefly discussed in the following sections.

#### 3.1.1. Functional Information Fields

**Figure 3:**
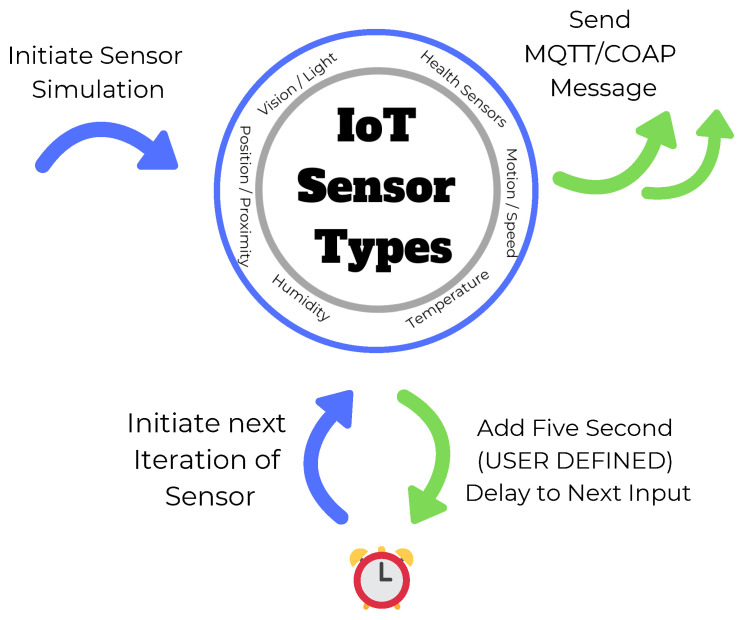
Time profile.

- **Device Type**—IoT-Flock [16,24] can create two types of devices: normal and malicious. The normal device is an ordinary device that sends or receives data or performs both operations simultaneously as defined by the user. Simultaneously, the malicious device is an attacking (harmful, malignant) device that can disturb the normal traffic flow of the use case.
- **Protocol**—IoT-Flock [16,24] supports two IoT application layer protocols, i.e., MQTT and COAP. In the MQTT device, the user will have to provide further details about the device like broker IP, username, password, subscribe, or publish topic. In the COAP device, the user will have to give information about the COAP server IP and the COAP command.
- **Data Profile**—The data profile of an IoT device illustrates the type of data that an IoT device can send. An IoT device can send either digital or analog data (values) attached with some notification message. The digital device will send binary values like on, off, or 1, 0. In contrast, the analog device will send the values in a given range appended with a user’s notification message. The notification message can either be a small text or an extensive text of megabytes.
- **Time Profile**—The time profile of an IoT device will prescribe the moment when the device will send the data as shown in Figure 3. The IoT-Flock [16,24] supports two kinds of time profiles entitled periodic or random. An IoT device with a regular time profile will transmit the data after a fixed interval of time as given by the user. In contrast, a device with a random time profile will send the data after some arbitrary interval within the random time range as specified by the user.

#### 3.1.2. Non-Functional Information Fields

The non-functional information of a device is also necessary to uniquely identify an IoT device. It includes the device name, device IP, and the number of devices. Device IP identifies the IoT device in traffic. Each device of use case will be assigned a separate IP which is used in communication.

The generation of IoT use cases can help researchers understand and meet the context-aware requirements of that specific domain. Moreover, use cases can also help researchers to analyze different dimensions like security requirements and others. IoT use cases developed in GUI mode of IoT-Flock [16,24] can be exported to XML format. IoT-Flock [16,24] development is done considering that IoT use case developed from any third party tool is provided in XML format can be run in IoT-Flock [16,24]. For this purpose, IoT console mode is introduced, where the user can simply provide XML file and complete IoT use case traffic generation will be started.

**Figure 4:**
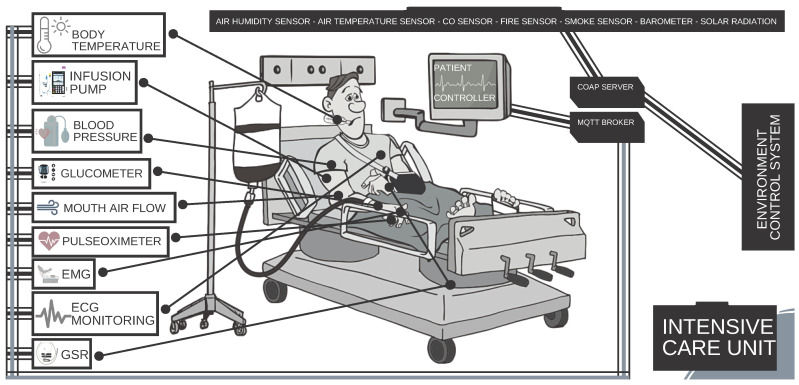
IoT ICU use case.

As discussed earlier in this work, we aimed to generate IoT traffic specifically for IoT-based healthcare system use cases. In this work, we considered IoT-based ICU use case. Therefore, we first need to create an IoT-based ICU use case using IoT-Flock [16,24]. For this purpose, we first analyzed the purpose, working principle, and type of data produced by the devices used commonly in an ICU, as shown in Figure 4. Thus, we created the IoT-based ICU use case using IoT-Flock [16,24] and added devices into it. Table 1 and Table 2 shows the list and the description of the devices we added to develop the IoT-based ICU in IoT-Flock [16,24]. The devices enlisted in Table 1 and Table 2.

The devices used in ICU use cases are classified into two categories, i.e., environmental monitoring devices and patient monitoring devices. The environment monitoring devices are used to monitor the environmental conditions of the ICU to maintain a good environment in the ICU. These devices are enlisted and described in Table 1. At the same time, the patient monitoring devices are mounted at particular parts of the body to observe the patient’s physical condition. The patient monitoring devices are used to continuously examine a patient’s physical condition to provide medical aid on the spot if the patient’s condition starts diminishing. These devices are enlisted and described in Table 2.

The IoT devices deployed in an ICU transmit a certain kind of data after a specific interval set by the ICU administrators. Therefore, we added two more characteristics of each device enlisted in Table 1 and Table 2. We called these characteristics data profile and time profile. In the data profile, we specified the type and range of data transmitted by each device, whereas, in the time profile, we set the time interval after which each device will send or receive the data. The data profile is defined by consulting each machine’s datasheet, whereas the time profile is determined based upon the authors’ general perception.

After the data profile and time profile of each device are specified, the next step is creating the device template using IoT-Flock [16,24]. Once the device template is created in IoT-Flock [16,24], it can add multiple times as it is or added after editing as required for a use case.

In our scenario, we used an IoT-based ICU with a capacity of 2 beds where each bed is equipped with nine patient monitoring devices (sensors) and one control unit. We called it as Bedx-Control-Unit where x depicts each bed’s number, i.e., Bed1 to Bed2. The Bedx-Control-Unit is responsible for taking specific actions like setting the time profile, the quantity of the dose given to the patient via an infusion pump, or generating the emergency alarm based upon the physical condition of the patient as observed by the patient monitoring devices.

Similarly, we added another control unit for environment monitoring devices and called it as Environment-Control-Unit. The Environment-Control-Unit is responsible for controlling the ICU environment conditions like keeping specific temperature, humidity level, detect smoke, and generating an emergency alarm in case of emergency conditions to maintain the required ICU environment. In our use case, both the patient monitoring and environment monitoring devices are MQTT-based devices. The MQTT protocol is connection-oriented and ensures that the packet is transmitted properly. Figure 4 illustrates the overall IoT-based ICU use case.

### 3.2. IoT Traffic Generation

We divided the testbed infrastructure into two networks to develop an extensive dataset, i.e., esteemed network and Invader network. The esteemed network is a standard IoT-based ICU network where the MQTT broker is deployed and multiple MQTT devices transmitting and receiving the data. The invader network is an attacking network that contains the attacking entities capable of originating the different types of attacks on targeted devices or servers.

#### 3.2.1. Normal Traffic Generation

We first designed an IoT-esteemed network for the dataset generation using the IoT-Flock [16,24] tool, which contains both patient and environment monitoring MQTT devices sending and receiving the network’s data under normal conditions. We created a use case of an IoT-based ICU with the capacity of 2 beds, where each bed is equipped with nine patient monitoring devices (i.e., sensors) and one control unit called as Bedx-Control-Unit. All these devices were created using the IoT-Flock [16,24] tool, which was running on a Linux machine. For each device, a virtual network interface was created by the IoT-Flock [16,24] tool on a single physical machine through which these devices were communicating. Moreover, the MQTT broker and the COAP server were running on a separate machine.

#### 3.2.2. Attack Generation

After the normal network started generating the IoT traffic, we created an invader network. The invader network includes ten attacking devices that are generating four types of attacks, including an MQTT distributed denial-of-service, MQTT publish flood, brute force, and SlowITE [37] attack. The following sections describe the types of attack that IoT-Flock [16,24] supports.

**Figure 5:**
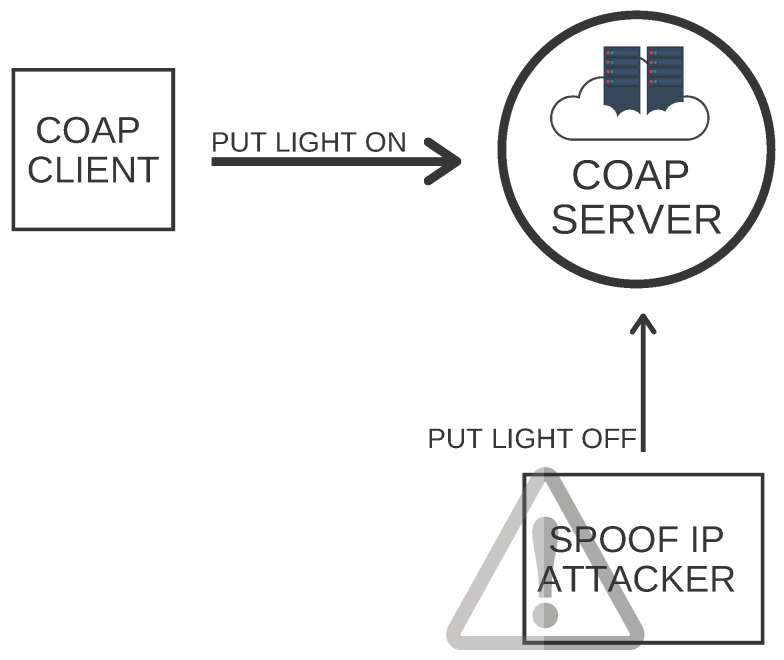
Constrained Application Protocol (COAP) replay attack.

- **MQTT Publish Flood**—DDoS attack can exhaust network bandwidth and victim system resources. Because of better mitigation techniques at the network and transport layer, DDoS attackers have now moved up the stack and are targeting application layer [38]. IoT devices follow the periodic or event-driven model for sending data using application layer protocols. The periodic model device sends data after every x interval, e.g., temperature sensor sends temperature data after every five seconds to the server. However, in event-driven model devices, it sends data when some event occurs, e.g., a motion sensor in the ICU can only send data to the server when it detects motion in the ICU. According to the authors of [39], MQTT publishing messages at high rate can cause a denial of service attack. Such attacks delay data transmission and are very harmful, specifically at industrial or other high levels like smart hospitals, smart transport systems, among others. The delay in data transmission can demolish these assets and be very harmful to human life.
- **MQTT Authentication Bypass Attack [40]**—To connect to the MQTT broker, which requires authentication, MQTT clients send MQTT to connect requests consists of username and password fields. It was discovered that MQTT authentication could be bypassed by eliminating the password field from the MQTT packet by only providing an existing username. Although this attack has been handled in the latest MQTT brokers, still an MQTT broker has to process this wrong packet, which can cause a delay in MQTT broker operations if sent in a large amount. Therefore, if the IPS blocks such an invalid packet, then the MQTT broker’s delaying issue can be prevented.
- **MQTT Packet Crafting Attack [41]**—In this attack, MQTT packets are specially crafted to crash an application. The attacker established a connection with the MQTT broker at the Transport layer and published it at the beginning instead of sending a connection request to the MQTT broker.
- **COAP Replay Attack**—In this attack, an intruder initially scans the network to get COAP client and server addresses and payload information. Then, the intruder changes the payload with incorrect data and sends it to the COAP server with spoofed COAP client IP. This attack’s severity can be seen in paper use cases where environment sensors use COAP protocols to transmit the surrounding data to the COAP server. For example, the temperature sensor is sending ICU temperature change, and, based on that value, the condition is set. If an attacker uses spoof IP, send an ICU temperature with some abnormal value and cause vulnerable and drastic damage in the ICU. An example of COAP replay attack is shown in Figure 5.

### 3.3. IoT Traffic Capturing

After the invader network and the esteemed network devices are created and started transmitting and receiving the data, the next step is to capture the packet flows and packets and payload. We used Wireshark [42] for capturing the packets. Wireshark [42] is a world-renowned and open-source network analysis tool that is used to capture the real-time packets and let you filter and inspect them. While capturing the packet flows, we developed a python utility to trace out the packet flows to extract the application layer features from the .pcap files.

Most of the cyber-attacks are application-layer attacks in the current era [43]. The traditional publicly available datasets consist of network and transport layer traffic details. Distributed Denial of Service (DDoS) attack at network and transport layer was known issue in the traditional network domain and researchers have contributed greatly to its defense [44].

According to Imperva Incapsula’s Global DDoS Threat Landscape 2017 Report [38], there has been a decrease in network layer attacks and an increase in application-layer attacks. Similarly, Kaspersky [45] reported that “the cream of cyber-criminal communities are now focusing on application layer DDoS attacks”. The protection against DDoS attacks in traditional application layer protocols like HTTP is under research [44]. However, there is no significant effort to defend IoT application layer protocols (COAP, MQTT) against DDoS attacks. The main reason behind the lack of attention is IoT datasets’ unavailability of IoT application layer attack traffic. To this aim, we developed a python utility to extract network and application layer features of IoT Traffic.

### 3.4. IoT Dataset Creation

We developed a python utility [46] that processes the pcap files and extracts features using the tshark library to extract the network and application layer features of IoT Traffic. We extracted network and application layer features from each generated pcap file and saved it into CSV with relevant traffic labels in this work. The features used are network layer features, application layer protocol-based features, and payload features, along with their description.

### 3.5. ML Model Development

The ML model development module consists of three major steps: dataset preprocessing, feature selection, ML models training, and testing. These steps are described in the subsequent sections.

#### 3.5.1. Dataset Pre-Processing

The dataset created after capturing the traffic was in the form of pcap files. To preprocess and analyze the created dataset, we converted the pcap files into CSV files using a python script. Furthermore, we substituted the categorical features of the dataset like protocol type (e.g., MQTT and COAP) with numerical values using Label Encoder to ease further processing. Finally, we authenticated the dataset to check the missing values. We found some missing values in the dataset and replaced them with 0. Afterward, the dataset was randomly split into train and test with a split ratio of 70:30, i.e., 70% of the dataset was randomly selected for training while 30% for testing.

#### 3.5.2. Features Selection

Feature selection plays a significant role in the performance of a machine learning model. After preprocessing the data, we applied a Logistic Regression (LR) algorithm due to its efficient implementation in the existing literature [3,18]. Using the LR algorithm, we selected the ten most significant features to train and test the machine learning models: [’frame.time_delta’, ’tcp.time_delta’, ’tcp.flags.ack’, ’tcp.flags.push’, ’tcp.flags.reset’, ’mqtt.hdrflags’, ’mqtt.msgtype’, ’mqtt.qos’, ’mqtt.retain’, ’mqtt.ver’]. The details of these features are given in [37].

#### 3.5.3. ML Models Training and Testing

When the data are processed and the features are selected, the next step is to train the machine learning model. Before training the machine learning models, we split the data into training and testing sets. For this purpose, we randomly split into train and test with a split ratio of 70:30, i.e., 70% of the dataset was randomly selected for training while 30% for testing. Afterward, we train six commonly used machine learning classifiers for malicious traffic detection in IoT healthcare environments using the training dataset. These six widely used machine learning classifiers include Naive Bayes (NB), K-Nearest Neighbors (KNN), Random Forest (RF), Adaboost (AB), Logistic Regression (LogR), and Decision Tree (DT) classifier. Finally, we test the trained models over the test set to evaluate each classifier’s performance to detect malicious traffic detection in IoT healthcare environments.

## 4. Results and Discussion

The machine learning classifiers’ performance is evaluated based on the four commonly used performance parameters: precision, recall, accuracy, and F1-score. These parameters are defined as follows.

**Precision:** is defined as the system’s ability to correctly detect the attack upon the occurrence of the security breach. It describes the ratio between the correctly predicted attacks (i.e., TP) and the actual results (i.e., TP + FP). Mathematically, it is described in Equation (1):

(1) $$\mathrm{Precision}=\frac{\mathrm{TP}}{\mathrm{TP}+\mathrm{FP}}\times 100$$

**Recall:** defines the ability of the system to correctly detect the botnet attack upon the occurrence of the attack in the network. Mathematically, it is expressed in Equation (2):

(2) $$\mathrm{Recall}=\frac{\mathrm{TN}}{\mathrm{TN}+\mathrm{FN}}\times 100$$

**Accuracy:** is defined as the ability of the system to correctly classify the attack packet as an “attack packet” and normal packet as a “normal packet”. It decribes the ratio of correct predictions with respect to all samples. Mathematically, it is expressed in Equation (3):

(3) $$\mathrm{Accuracy}=\frac{\mathrm{TP}+\mathrm{TN}}{\mathrm{TP}+\mathrm{FN}+\mathrm{TN}+\mathrm{FP}}\times 100$$

**F1-score:** is the harmonic mean of precision and recall. It describes the ratio of correct predictions in test set for both normal and attack traffic. Mathematically, it is expressed in Equation (4):

(4) $$F1-\mathrm{score}=2\times \frac{\mathrm{Precision}*\mathrm{Recall}}{\mathrm{Precision}+\mathrm{Recall}}$$

We need the confusion matrix of each machine learning classifier to calculate the performance mentioned above parameters. Further, we also need to define the following terms:

- **True Positive (TP) :** The ML model truly predicted the attack flow as an attack.
- **True Negative (TN) :** The ML model truly predicted the normal flow as normal.
- **False Positive (FP) :** The ML model wrongly predicted the normal flow as an attack.
- **False Negative (FN) :** The ML model wrongly predicted the attack flow as normal.

Table 3, Table 4, Table 5, Table 6, Table 7 and Table 8 show the confusion matrices of each individual ML classifier, obtained for malicious traffic detection over IoT healthcare test dataset generated using IoT-Flock [16,24]. Based on these confusion matrices, we evaluated each ML classifier’s performance using the above parameters’ performance. Finally, in Table 9, we enlisted the performance evaluation results of ML classifiers test for both the normal and malicious traffic detection over test-set extracted from the IoT healthcare dataset that we generated using IoT-Flock [16,24].

**Figure 6:**
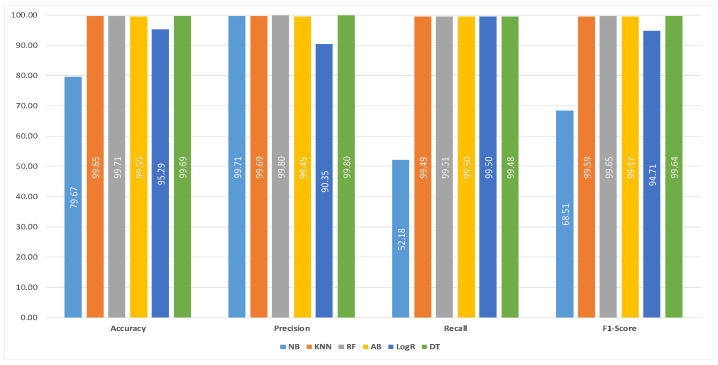
Performance comparison of six commonly used machine learning classifiers for malicious traffic detection over IoT healthcare dataset.

Figure 6 illustrates the performance comparison of all six ML classifiers for detecting the normal and malicious traffic in the IoT healthcare environment. It can be observed that among the six ML classifiers, the RF classifier outperformed all other classifiers with 99.7068% precision, 99.7952% recall, 99.5123% accuracy, and 99.6535% F1-score.

Besides the above-discussed performance matrices, we also evaluated the performance of all the six ML classifiers by visualizing the area under the receiver operating characteristics (ROC) curve. The ROC curve is created by plotting the true positive rate (TPR) on the y-axis and false positive rate (FPR) on the x-axis at different threshold values.

- The TPR is a ratio between the correctly predicted attacks (i.e., TP) and all the actual attack samples (i.e., TP + FN). Mathematically, it is written as Equation (5):
  (5) $$\mathrm{True}\;\mathrm{Positive}\;\mathrm{Rate}\left(\mathrm{TPR}\right)=\frac{\mathrm{TP}}{\mathrm{TP}+\mathrm{FN}}\times 100$$
- The FPR is defined as the ratio between wrong attack detection (i.e., FP) and all the normal samples (i.e., FP + TN). Mathematically, it is described in Equation (6):
  (6) $$\mathrm{False}\;\mathrm{Positive}\;\mathrm{Rate}\left(\mathrm{FPR}\right)=\frac{\mathrm{FP}}{\mathrm{FP}+\mathrm{TN}}\times 100$$

**Figure 7:**
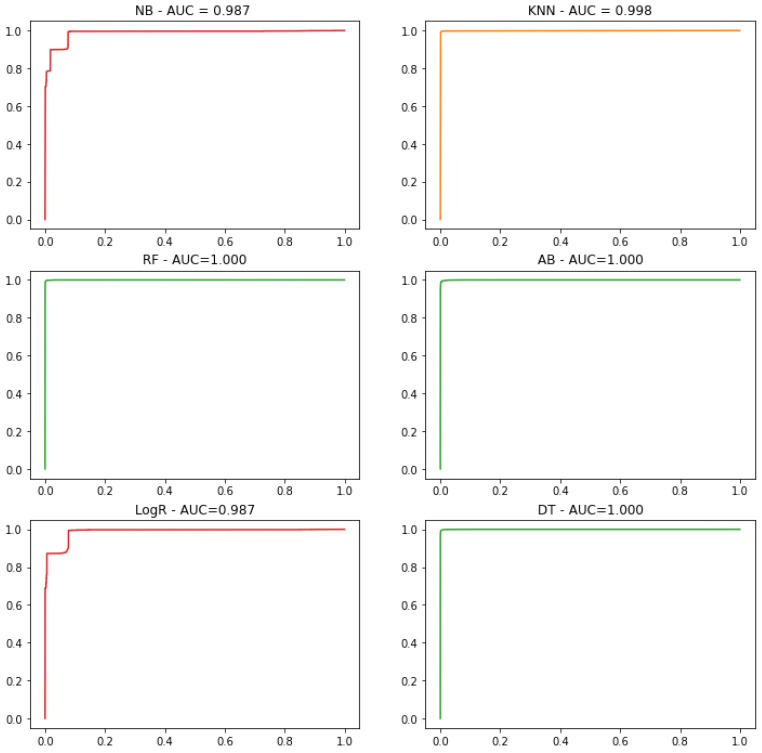
ROC curves and Area Under the ROC Curve (AUC) of six commonly used machine learning classifiers for detecting the malicious in IoT ICU use case.

Figure 7 shows the ROC curves of all six ML classifiers used for detecting the malicious traffic in IoT ICU use case. The area under the ROC curve is also mentioned in Figure 7, by which one can easily analyze the performance of each classifier for efficiently detecting the malicious attacks. As can be observed in Figure 7, the AUC for the RF, AB, and DT classifier, is 1, which means that these three classifiers are much efficient in truly detecting attacker attempts in the IoT ICU use case as compared to the other ML classifiers.

The experiments demonstrate how the proposed framework can detect cyber-attacks to secure and protect the IoT healthcare environment from cyber-attacks. By following the proposed framework’s key steps as illustrated in Figure 1, one can quickly develop AI-based security solutions for any other IoT use case. Furthermore, the experimental results and dataset generated are also helpful for developing context-aware IoT security solutions, especially for the IoT healthcare environment. The dataset generated in the current study can be shared with the other researchers for further experimentation based on their request.

## 5. Conclusions

The rapid advancement of IoT technology has focused researchers’ and technologists’ attention on the design of IoT healthcare systems. Many IoT healthcare systems have been proposed in recent years, but these systems endure the security backdoor. IoT healthcare systems’ security is crucial as any security breach or cyber-attack in such systems may cause a rigorous effect on human life and even may cause death in some instances. Therefore, in this work, we proposed a framework for developing IoT context-aware security solutions to detect malicious traffic in IoT healthcare environments. The proposed framework is composed of an IoT traffic generator tool in which an IoT-based ICU use case is created to generate standard and malicious traffic. The generated traffic is then converted into a dataset by extracting the features using a python script. Afterwards, we trained and test six commonly used machine learning (ML) classifiers over the generated dataset for malicious and traditional traffic detection in the IoT healthcare environment. Finally, we test and analyzed the performance of each trained ML classifier. Among the six ML classifiers, the Random Forest classifier performed the best with 99.7068% precision, 99.7952% recall, 99.5123% accuracy, and 99.6535% F1-score. The experimental results demonstrate the effectiveness of the proposed framework for developing efficient IoT context-aware security solutions. Moreover, the proposed framework and generated dataset are helpful for the researchers to pursue the proposed method for developing more robust context-aware security solutions, especially for IoT healthcare environments. Furthermore, with the proposed framework’s help, the researchers can quickly generate the traffic of other IoT use cases in order to develop AI-based security solutions for other IoT use cases.

## Figures and Tables

**Table 1:** Environment monitoring sensors.

Device Name	Description	Data Profile	Time Profile
Air Humidity Sensor	Measures the air humidity	0–100% Rh (Relative Humidity)	5 s
Air Temperature Sensor	Measures the air temperature	−20–70 °C	5 s
CO Sensor	Measures the concentration of CO gas in Environment	0–2000 ppm	2 s
Fire Sensor	Detects the presence of fire and flame	Flame detected (0,1)	2 s
Smoke Sensor	Detects the smoke in the air	300–10,000 ppm	2 s
Barometer	Measures the atmospheric pressure	800–110 hPa	5 s
Solar Radiation Sensor	Measures the direct solar radiations by thermopile	Spectrum of radiation (0–2000 Watt/m^2^)	5 s

**Table 2:** Patient monitoring sensors.

Device Name	Description	Data Profile	Time Profile
Remote Electrocardiogram (ECG) monitoring	Test the electrical and muscular functions of the heart	Pulse Rate (0–200 bpm)	1 s
Infusion Pump	A generic device used to deliver the nutrients and drugs to patient at a controlled amount.	Dose (10–100 mL)	10 min
Pulsoximeter (SPO2)	A device that tells the oxygen saturation (i.e., amount of oxygen dissolved) in blood	Oxygen in blood (35–100%)	1 s
Nasal/Mouth AirFlow Sensor	Provides the (breathing) respiratory rate of a patient	Device Respiratory rate (0–60 ppm peaks/min)	1 s
Blood pressure monitor Sensor	Measure the pressure of the blood in the arteries when heart beats.	systolic & diastolic pressure (0–300 mm Hg)	2 s
Glucometer	A device used to determine the amount of glucose in the blood.	Glucose in Blood (10–150 mg/dL)	10 min
Body Temperature Sensor	Measures the temperature of the body	Temperature (0–120 F)	10 min
Electro- myography (EMG) Sensor	Measures the electric potential produced by the body muscles	Muscle rate (0–60 cpm) (contractions/min)	5 min
Galvanic skin response (GSR) Sensor	Measures the electrical conductance of skin.	Conductance (0–20 uS) (micro Semens)	5 min

**Table 3:** Confusion matrix of Naive Bayes (NB) classifier test for malicious and normal traffic detection over IoT healthcare dataset generated using IoT-Flock [16,24].

NB Classifier		Predicted	
		**Normal**	**Attack**
Actual	**Normal**	32,583	37
	**Attack**	11,471	12,518

**Table 4:** Confusion matrix of K-Nearest Neighbors (KNN) classifier test for malicious and normal traffic detection over IoT healthcare dataset generated using IoT-Flock [16,24].

KNN Classifier		Predicted	
		**Normal**	**Attack**
Actual	**Normal**	32,545	75
	**Attack**	123	23,866

**Table 5:** Confusion matrix of Random Forest (RF) classifier test for malicious and normal traffic detection over IoT healthcare dataset generated using IoT-Flock [16,24].

RF Classifier		Predicted	
		**Normal**	**Attack**
Actual	**Normal**	32,571	49
	**Attack**	117	23,872

**Table 6:** Confusion Matrix of Adaboost (AB) Classifier Test for malicious and normal traffic detection over IoT healthcare dataset generated using IoT-Flock [16,24].

AB Classifier		Predicted	
		**Normal**	**Attack**
Actual	**Normal**	32,487	133
	**Attack**	119	23,870

**Table 7:** Confusion matrix of Logistic Regression (LogR) Classifier test for malicious and normal traffic detection over IoT healthcare dataset generated using IoT-Flock [16,24].

LogR Classifier		Predicted	
		**Normal**	**Attack**
Actual	**Normal**	30,071	2549
	**Attack**	119	23,870

**Table 8:** Confusion matrix of Decision Tree (DT) classifier test for malicious and normal traffic detection over IoT healthcare dataset generated using IoT-Flock [16,24].

DT Classifier		Predicted	
		**Normal**	**Attack**
Actual	**Normal**	32,572	48
	**Attack**	125	23,864

**Table 9:** Performance evaluation of six commonly used machine learning classifiers test for malicious and normal traffic detection over IoT healthcare dataset generated using IoT-Flock [16,24].

ML Classifier	Precision	Recall	Accuracy	F1-Score
NB	79.6711	99.7053	52.1823	68.5092
KNN	99.6502	99.6867	99.4873	99.5869
RF	99.7068	99.7952	99.5123	99.6535
AB	99.5548	99.4459	99.5039	99.4749
LogR	95.287	90.3516	99.5039	94.7072
DT	99.6944	99.7993	99.4789	99.6388

## Data Availability

Not applicable.
